# Theoretical and Computational Insight into Solvent and Specific Ion Effects for Polyelectrolytes: The Importance of Local Molecular Interactions

**DOI:** 10.3390/molecules25071661

**Published:** 2020-04-03

**Authors:** Jens Smiatek

**Affiliations:** Institute for Computational Physics, University of Stuttgart, Allmandring 3, D-70569 Stuttgart, Germany; smiatek@icp.uni-stuttgart.de

**Keywords:** polyelectrolyte solutions, solvents, specific ion effects, molecular interactions, molecular theory of solutions

## Abstract

Polyelectrolytes in solution show a broad plethora of interesting effects. In this short review article, we focus on recent theoretical and computational findings regarding specific ion and solvent effects and their impact on the polyelectrolyte behavior. In contrast to standard mean field descriptions, the properties of polyelectrolytes are significantly influenced by crucial interactions with the solvent, co-solvent and ion species. The corresponding experimental and simulation results reveal a significant deviation from theoretical predictions, which also highlights the importance of charge transfer, dispersion and polarization interactions in combination with solvation mechanisms. We discuss recent theoretical and computational findings in addition to novel approaches which help broaden the applicability of simple mean field theories.

## 1. Introduction

Polyelectrolytes are charged macromolecules with a substantial amount of ionic or ionizable groups [1]. In contrast to uncharged polymers, the conformational arrangement and the resulting charge state of polyelectrolytes are governed by long-range electrostatic interactions which complicate the development of theoretical descriptions [2,3,4,5]. Besides the predominant electrostatically driven mechanisms, recent simulation and experimental studies have shown that also the local interactions between the polyelectrolyte, the solvent molecules and the ions induce a broad plethora of interesting effects. Thus, it was observed that the properties of the polyelectrolyte depend crucially on the counterion distribution, the fraction of polar and apolar groups, the position of charges as well as the properties of the solution. Moreover, also the presence of external electric fields, multicomponent solutions or complex geometries enforce significant changes when compared to recent outcomes for polyelectrolytes in aqueous and homogeneous bulk solution [6,7,8,9,10,11,12,13,14,15]. With regard to these specific molecular interactions, it becomes clear that a straightforward and reliable mean field description for polyelectrolytes which takes these contributions into consideration is far out of reach.

Nowadays, computer simulations in combination with refined experimental techniques help to fill the aforementioned knowledge gaps. In more detail, various coarse-grained and atomistic models in combination with molecular dynamics (MD) or Monte Carlo simulations are often used to study the dynamic and structural properties of polyelectrolytes at distinct time and length scales [7,8,16,17]. For the study of short-range molecular behavior, one usually employs atomistic MD simulations, providing a high level of detail and accuracy for short time and length scales [15,16,18,19,20]. When compared to atomistic models, coarse-grained approaches simplify or even ignore molecular and chemical details for the sake of computational efficiency [16,21,22,23]. In consequence, a large portion of local molecular interactions is not sufficiently taken into consideration, which questions the applicability of these approaches for the study of meaningful short-range properties. However, despite their limitations in terms of detailed molecular interactions, coarse-grained models provide a reasonable level of accuracy for the analysis of polyelectrolyte dynamics at large length and long time scales [7,17].

Over the last years, specifically solvent- and ion-related effects for polyelectrolytes attracted considerable attention. For instance, previous atomistic MD simulations revealed that the molecular solvation behavior influences significantly the amount of dissociated counterions, and thus the corresponding conformational behavior with regard to repulsive electrostatic interactions or charge screening effects along the polyelectrolyte backbone [18,24,25]. The molecular arrangement of the solvent molecules is influenced by the presence of the polyelectrolyte as manifested by local variations of the dielectric constant, charge hydration asymmetry effects or modified charge transport mechanisms [26,27,28]. Comparable molecular interactions also dominate the occurrence of various polyelectrolyte structures in bulk phase, as can be seen by the formation of polyelectrolyte micelles, pearl-necklace structures, the onset of microphase separation processes between polar and apolar regions as well as the formation of polyelectrolyte complexes [29,30,31,32,33,34] and coacervates [35]. With regard to the broad plethora of observations and applications, it becomes clear that a more fundamental understanding of local interactions and short-range effects on polyelectrolytes is of urgent need. In this short review article, we shed more light on solvent- and ion-related effects which govern the properties of polyelectrolytes in bulk solutions.

The article is organized as follows. In the next section, we will briefly summarize basic principle of standard approaches like electrostatic screening and counterion condensation theories. In the third section, we will discuss recent findings in accordance with the influence of various solvents on the polyelectrolyte behavior. Hereafter, it will be highlighted how specific ion effects govern the structural properties of polyelectrolytes. All outcomes reveal significant deviations from mean field descriptions and we will shed more light on novel theoretical approaches. In the last section, we will summarize and discuss the main outcomes.

## 2. Theoretical Background: Polyelectrolytes and Ions in Solution

As already mentioned, the structural properties of polyeletrolytes are governed by a combination of electrostatic and short-range molecular interactions. With regard to this point, the influence of dissociated counterions as well as salt ions have to be considered as integral contribution to the observed polyelectrolyte behavior. Here, we discuss the theoretical description of electrostatic effects in terms of counterion condensation and charge screening mechanisms.

### 2.1. Electrostatic Screening Effects

Charged species in solution are governed by electrostatic interactions. For two charged and small species in a solvent with dielectric constant $\epsilon_{r}$, the electrostatic Coulomb potential Φ(r) shows a long-range decay

(1) $$\Phi \left(r\right)∼\frac{1}{\epsilon_{r}r}$$

where *r* denotes the distance between the charges at a high dilution. At moderate and high ion concentration, the long-range decay of Equation (1) changes significantly due to electrostatic screening effects. A suitable mathematical description for polyelectrolytes and ions of species α with valency $z_{\alpha }$ and unit charge *e* is given by the mean field Poisson-Boltzmann (PB) equation [36,37,38]

(2) $$\frac{\partial^{2}}{\partial r^{2}}\Phi \left(r\right)=-\sum_{\alpha }\frac{z_{\alpha }e}{\epsilon_{r}\epsilon_{0}}\rho_{\alpha }^{\infty }\exp\left(-\frac{z_{\alpha }e\Phi \left(r\right)}{k_{B}T}\right)$$

with Boltzmann constant $k_{B}$, temperature *T*, and vacuum dielectric constant $\epsilon_{0}$, where $\rho_{\alpha }^{\infty }$ corresponds to the ion density in bulk phase at Φ(r)=0. A well-known approximation for Equation (2) can be introduced for simple and diluted ions under the condition of charge neutrality $\left(\sum_{\alpha }z_{\alpha }\rho_{\alpha }^{\infty }=0\right)$, unit valency *z* and moderate maximum electrostatic potential $\Phi \left(r_{s}\right)=\Phi_{s}$ at the hydrodynamic boundary position $r_{s}$ with $\Phi_{s}/k_{B}T\ll 1$. With regard to the latter condition, Equation (2) can be linearized, which yields for the electrostatic potential around a spherical object in presence of monovalent ions

(3) $$\Phi \left(r\right)∼\frac{1}{\epsilon_{r}r}e^{-\kappa_{D}r}$$

with the Debye-Hückel length

(4) $$\kappa_{D}^{-1}=\sqrt{\frac{\epsilon_{r}\epsilon_{0}k_{B}T}{2e^{2}\rho^{\infty }}}$$

thereby highlighting a short-range decay of electrostatic interactions at finite salt concentration. Thus, it becomes clear that the ion density, the temperature and the dielectric constant as parameters of the solution have a significant influence on the polyelectrolyte behavior. In terms of a mechanistic explanation, an ion cloud around the polyelectrolyte forms due to attractive Coulomb interactions, which thus lowers the effective charge in terms of electrostatic screening effects. It was recently shown that the corresponding charge screening mechanisms also govern the dynamic properties of polyelectrolytes [6,39,40,41,42]. In more detail, for a polyelectrolyte with mean radius of gyration $R_{g}$, a screening of hydrodynamic interactions can be observed for $R_{g}\gg 1/\kappa_{D}$ whereas a more standard polymer-like behavior becomes obvious for $R_{g}\leq 1/\kappa_{D}$ [10,11]. The corresponding PB equation as well as the Debye-Hückel approach are typical examples of mean field theories. More specifically, all ion sizes and correlations as well as excluded volume effects are ignored such that only the most dominating conributions are taken into consideration by simplification.

### 2.2. Counterion Condensation Theory

For strong polyelectrolytes with a high charge fraction, the full dissociation of counterions is reduced by electrostatic attraction in terms of condensed counterions. The Manning-Oosawa counterion condensation (MOCC) theory [43,44,45] aims to estimate the number of condensed counterions by introducing a very long and charged cylinder which mimics the properties of an idealized polyelectrolyte. For the sake of mathematical simplicity, all ionic correlations, finite ion sizes as well as the presence of explicit solvent molecules are ignored. In principle, counterion condensation mechanisms rely on a combination of strong electrostatic interactions between the counterions and the polyelectrolyte and the translational entropy of the free counterions [46,47,48,49]. The loss of the translation entropy for the counterions upon condensation is compensated by electrostatic attraction which rationalizes the fact that only highly charged polyelectrolytes reveal counterion condensation behavior. Moreover, in very dilute polyelectrolyte solutions, the entropic penalty for counterion condensation is very high, such that free counterions dominate. However, with increasing polyelectrolyte concentration, the entropic loss for counterion condensation decreases, such that a finite number of condensed counterions can be observed [48]. Noteworthy, one should also consider the solvation entropy and enthalpy between the counterions and the solvent but these contributions are usually neglected for the sake of simplicity. Further approaches also take the polyelectrolyte flexibility as well as the polarity into consideration [46,47,48]. However, in terms of a straightforward mean field consideration which ignores all specific intra- and intermolecular interactions, the stable fraction of condensed counterions is determined at the threshold where the derivative of the resulting free energy with respect to the amount of condensed counterions vanishes [49]. Hence, the central quantity in the MOCC theory is the so-called Manning parameter

(5) $$\xi =\frac{\lambda_{B}}{b}$$

with the Bjerrum length

(6) $$\lambda_{B}=\frac{e^{2}}{4\pi \epsilon_{0}\epsilon_{r}k_{B}T}$$

and the contour charge length *b*, which is the distance between two charged groups of the polyelectrolyte. The value of the Bjerrum length corresponds to the distance between the charges, where electrostatic interactions are of comparable magnitude as the thermal energy $k_{B}T$. In accordance with the theory [44], strong counterion condensation sets in for values ξ≥1, a condition which is met for polyelectrolytes with small *b* and solvents with large $\lambda_{B}$ (Equation (6)). Thus, even at very dilute or vanishing salt concentrations, electrostatic interactions between the ions can be ignored for distances $r\geq \lambda_{B}$, as induced by *dielectric screening* mechanisms of the surrounding solvent molecules [34,50].

An explicit expression for the number of condensed counterions can be derived as follows [44]. With regard to the Debye-Hückel approach (Equation (3)), the electrostatic interactions and thus the electrostatic work to assemble a linear polyelectrolyte with *P* monomers of charge *q* reads [51]

(7) $$w_{\mathrm{el}}=-q^{2}\xi P\ln\left(\kappa_{D}b\right)$$

which corresponds to a very long and linear polyelectrolyte with fixed charge fraction. Thereby, a mean field description of the linearized PB equation is taken into consideration, which also points to the fact that all ionic correlations as well as excluded-volume and finite size effects of the ions are ignored. Due to the presence of counterions, the reduced charge of a monomer reads

(8) $$q_{\mathrm{eff}}=\left(1-\theta \right)q$$

with 0≤θ≤1 as a consequence of the fraction θ of condensed counterions. The effective work overcoming the loss of translational entropy for the counterions required to assemble the effective charge on the polyelectrolyte thus reads [51]

(9) $$\frac{w_{\mathrm{el}}}{k_{B}T}=-{\left(1-\theta \right)}^{2}\xi P\ln\left(\kappa_{D}b\right)$$

after insertion of Equation (8). In presence of a monovalent and 1:1 electrolyte salt with bulk concentration $c_{s}$, the work required to transfer θ counterions from bulk solution to the polyelectrolyte [51] is given by

(10) $$\frac{w_{\mathrm{tr}}}{k_{B}T}=\theta P\ln\left(\frac{c_{1}\theta }{c_{s}Z\left(\theta \right)}\right)$$

with the partition function Z(θ) for the condensed counterions, and with the concentration of one uncondensed counterion $c_{1}$ as reference state. As a sum of both contributions, the total work required for the formation of the polyelectrolyte reads $w=w_{\mathrm{tr}}+w_{\mathrm{el}}$ which separates the work into ion- and polyelectrolyte-related contributions. If a steady equilibrium distribution of the ions around the polyelectrolyte is assumed, the fraction of condensed counterions can be calculated by

(11) $$\frac{\partial w}{\partial \theta }=0$$

which yields

(12) $$P^{-1}\frac{\partial w}{\partial \theta }=-\xi k_{B}T\left[\theta -\left(1-\xi^{-1}\right)\right]\ln\left(c_{s}/c_{1}\right)+f\left(\theta \right)$$

where f(θ) does not depend on the salt concentration.

With regard to Equation (12), one can define two limiting cases. For ξ≤1, it follows that the equilibrium state of minimum free energy is located at θ=0 which corresponds to free counterions only. In contrast for ξ>1, it follows that Equation (12) changes sign at $\theta =1-\xi^{-1}$, which corresponds to the actual minimum free energy state and thus highlights the presence of non-vanishing condensed counterions. Hence, the actual stable fraction of condensed counterions reads

(13) $$\theta =1-\left(\frac{1}{\xi }\right)$$

which implies that counterion condensation is initiated by small values of contour charge lengths as well as high values for the Bjerrum length in accordance with Equation (5). In summary, all mean field approaches ignore electrostatic correlations between the ions as well as finite size and excluded volume effects for the sake of simplicity. Moreover, long range electrostatic interactions are replaced by short range counterion screening interactions which emphasizes the mean field characteristics of the previous approach. Despite all crucial approximations, the validity of the MOCC theory was demonstrated for coarse grained polyelectrolyte and counterions in a continuum solvent [52].

A more advanced theory, focusing on an explicit expression for the radial counterion density around the polyelectrolyte is represented by the PB cell model approach [52,53]. Here, the electrostatic potential is computed around a charged rod, which corresponds to a very long cylinder with radius $r_{0}$, thereby resembling an idealized polyelectrolyte with uniform charge distribution. Moreover, it is assumed that the rod is embedded in a cylindrical cell with a fixed and finite radius. With regard to these considerations, the PB equation (Equation (2)) is transformed to cylindrical coordinates in order to evaluate the charge distribution of monovalent counterions in terms of the Debye-Hückel approach (Equation (4)) around the rod. In combination with boundary conditions on the derivative of the electrostatic potential, two equations can be obtained, which can be used to define an expression for the Manning radius $R_{M}$ and a prefactor $\gamma_{M}$. The Manning radius thus defines the largest distance for condensed counterions such that the radial fraction of condensed counterions is given by

(14) $$\theta \left(r\right)=1-\left(\frac{1}{\xi }\right)+\frac{\gamma_{M}}{\xi }\tan\gamma_{M}\ln\left(\frac{r}{R_{M}}\right)$$

which coincides for $r=R_{M}$ with Equation (13).

Despite the reasonable assumptions of the MOCC theory and its modifications, recent atomistic MD simulations highlighted significant deviations to the cell model approach for short distances around polyelectrolytes [15,18,54,55]. In order to correct for these deviations, it was suggested [54] to introduce a modified Poisson-Boltzmann equation with $\psi_{\infty }=0$ according to

(15) $$\frac{\partial^{2}}{\partial r^{2}}\psi \left(r\right)=-\sum_{\alpha }\frac{z_{\alpha }e}{\epsilon_{r}\epsilon_{0}}\rho_{\alpha }^{\infty }\exp\left(-z_{\alpha }e\frac{\left(\phi \left(r\right)+V_{s}\left(r\right)\right)}{k_{B}T}\right)$$

with the additional potential

(16) $$V_{s}\left(r\right)=V_{0}\exp\left(-\frac{{\left(r-r_{0}\right)}^{2}}{\sigma_{s}^{2}}\right)$$

where the prefactor $V_{0}$ can be interpreted as an empirical hydration potential, with $r_{0}$ as the corresponding position of the first counterion shell around the polyelectrolyte and $\sigma_{s}$ as the range of ion-specific interactions [54]. With regard to the contribution of the hydration potential, it becomes clear that the solvent has a significant influence on the counterion distribution as well as on the amount of condensed counterions [15].

In summary, it can be concluded that mean field descriptions mainly rely on continuum solvent approaches with fixed values for the dielectric constant as well as crucial approximations for the ions and the polyelectrolyte, such that any molecular or local interactions between the species are ignored.

## 3. Solvent Effects

In contrast to previous mean field considerations as discussed in the last section, it was shown that specifically the molecular interactions between the polyelectrolyte, the ions as well as the solvent molecules play a crucial role [34]. In this section, we will concentrate on the influence of solvent-related contributions in more detail. Thereby, it can be shown that standard mean field theories like the MOCC approach are not fully sufficient to describe all relevant effects. As it was discussed previously, underlying principles rely mainly on electrostatic interactions as well as counterion translational entropy effects. Further important contributions which are usually ignored can be attributed to translational and orientational entropy effects of the solvent molecules as well as specific molecular interactions in terms of short range electronic and polarization effects. Due to the complexity of these mechanisms, such approaches are usually neglected in standard mean field theories which questions the validity of such models for a more reliable interpretation of the observed effects.

### 3.1. Dielectric Decrement Effects

As already mentioned, the solvent changes the strength of electrostatic interactions between the charged groups of the polyelectrolyte and the ions. Vice versa, structural properties of the solvent like the molecular arrangement as well as the dynamic behavior are also affected by the presence of charged species [56]. These effects become more pronounced for small polar solvent molecules like water as it becomes obvious by charge hydration asymmetry effects around positively and negatively charged ions [57]. In consequence, one usually observes a locally varying dielectric constant in close vicinity of charged species. In addition to linear respone relations [19,20,58,59], a simple expression for the global dielectric constant $\epsilon_{r}$ reads [60,61,62]

(17) $$\epsilon_{r}=1+4\pi \frac{\langle M_{\mathrm{trans}}^{2}\rangle }{3Vk_{B}T}$$

with the time-averaged squared net total translational dipole moment $\langle M_{\mathrm{trans}}^{2}\rangle$ of the solvent molecules in the respective volume *V*. With regard to Equation (17), it becomes clear that any significant change of the local net total translational dipole moment due to the presence of charged species or interfaces at distance *r* results in a locally varying dielectric constant in accordance with $\langle M_{\mathrm{trans}}^{2}\left(r\right)\rangle ∼\left(\epsilon_{r}\left(r\right)-1\right)$. Corresponding simulation findings for water molecules around charged and uncharged objects as well as around polyelectrolytes were observed in Refs. [26,63,64,65,66]. All studies reveal significant ordering effects when compared to bulk phase due to the polarity of water molecules. Hence, and in agreement with experimental findings, a reasonable assumption for highly polar solvents like water due to charge-induced ordering effects reads $\langle M_{\mathrm{trans}}^{2}\left(r\right)\rangle <\langle M_{\mathrm{trans}}^{2}\rangle$ which reveals a decreased local dielectric constant in presence of charged species when compared to pure solvents [34,50]. In consequence, the local dielectric constant is lower when compared to the bulk dielectric constant in accordance with $\epsilon_{r}\left(r\right)<\epsilon$, which means that electrostatic effects become more pronounced for polar solvents in close distance to charged interfaces.

Noteworthy, recent simulations provided more detailed insights into the mechanisms behind these so-called dielectric decrement effects. For low salt concentrations, it was shown that the local change of $\epsilon_{r}\left(r\right)$ at moderate salt concentrations is mainly affected by solvent-solvent instead of ion-solvent or ion-ion interactions [19]. The authors of the corresponding study provided a decomposition of the dielectric spectrum for a water-dimethyl sulfoxide mixture in presence of dilute salt conditions which shows that ion-ion as well as ion-solvent interactions are of minor importance. The proposed method is broadly applicable also for other mixtures and relies on the evaluation of the autocorrelation function for the ionic conductivities. A more detailed introduction can be found in Refs. [19,56]. In other words, the influence of ions or charged groups on solvent molecules in the first solvation shell (contact solvent molecules) also affects the distribution and orientation of solvent molecules in the second and higher solvation shells. These findings are in line with Equation (17) and provide a rationale for the observation that polar and protic solvents with a more pronounced internal structure show stronger dielectric decrement effects when compared to more apolar media.

In terms of a phenomenological explanation for dielectric decrement effects, the electrostatic field around the ions E(r) perturbs the relaxation of the surrounding solvent molecules in terms of multipole interactions according to [34,50]

(18) $$\epsilon_{r}\left(r\right)=\epsilon_{r}+\beta E{\left(r\right)}^{2}$$

where β<0 denotes a solvent- and concentration-specific coupling constant. Beyond a critical salt concentration, it can be shown that all solvent molecules are part of the first or second solvation shell around the ions which rationalizes global changes in the bulk dielectric constant as discussed in Refs. [19,33,66,67,68]. In these publications, it was shown that the corresponding methodology also holds for coarse-grained simulations as well as for simple and complex ions at different concentrations. Interestingly, although the corresponding expressions (Equation (18)) can be used for further evaluation in mean field PB approaches, their occurrence can be solely attributed to specific molecular representations which are usually ignored.

**Figure 1:**
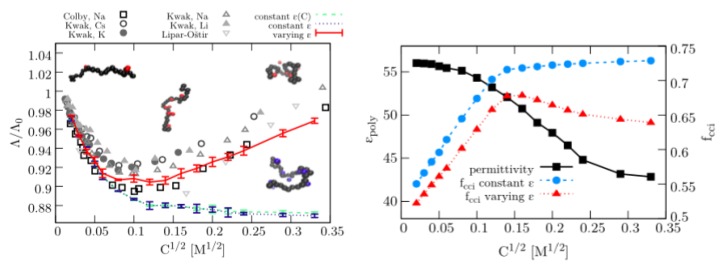
(**Left**) Normalized ionic conductivity $\Lambda /\Lambda_{0}$ of polyelectrolyte solutions with varying salt concentrations $C^{1/2}$. The single dots denote the values of experimental outcomes. The straight solid red line shows the results of coarse-grained molecular dynamics simulations with a varying dielectric constant. The dashed green line highlights the corresponding results for a constant value of $\epsilon_{r}$. Snapshots of polyelectrolyte conformations for specific salt concentrations in combination with counterions are shown in the inset. (**Right**) Fraction of condensed counterions $f_{\mathrm{cci}}$ around a highly charged polyelectrolyte for constant (blue circles) and varying values of the dielectric constant $\epsilon_{\mathrm{poly}}$ (red triangles). A foxed dielectric constant was set to a value of $\epsilon_{r}=56$ whereas the resulting outcomes in terms of dielectric decrement effects are denoted as black triangles. Figure reproduced from Ref. [65].

Recent experimental and simulation outcomes suggested that comparable effects also rationalize the dynamic properties of polyelectrolytes in presence of high salt concentrations [26,65,69]. Specifically the dielectric decrement effect and its influence on the polyelectrolyte in terms of dynamic properties like ionic conductivities attracted recent experimental and theoretical attention [26,65]. In these publications, the authors used coarse-grained Lattice-Boltzmann/MD simulations in combination with advanced electrostatic solvers to study the influence of polyelectrolytes and ions on the dielectric constant of the solution. In terms of the ionic conductivity, the corresponding simulations helped to unravel previous experimental findings, where it was observed that the ionic conductivity decreases with increasing salt concentrations until a certain threshold where it increases again. Thus, it was observed that the conductivity of polyelectrolyte solutions shows a non-linear change with higher salt concentrations. The corresponding findings are depicted in Figure 1.

The results for the dielectric constant $\epsilon_{r}$ and the fraction of condensed counterions $f_{\mathrm{ccl}}$ reveal that the dielectric constant decreases from $\epsilon_{r}=56$ at a square-root salt concentration of $C^{1/2}=0.01$ M${}^{1/2}$ to a minimum value of $\epsilon_{r}=42$ at $C^{1/2}=0.34$ M${}^{1/2}$. In combination, a non-linear change of the normalized ionic conductivity $\Lambda /\Lambda_{0}$ can be observed at $C^{1/2}=0.13$ M${}^{1/2}$. The corresponding fraction of counterions $f_{\mathrm{cc}}$ is highest at $C^{1/2}=0.1-0.15$ M${}^{1/2}$ with $f_{\mathrm{cc}}=0.68$ which coincides with the fact that the ionic conductivity is lowest for these values. At higher salt concentrations, the amount of condensed counterions decreases to $f_{\mathrm{ccl}}=0.64$ which rationalizes an increasing ionic conductivity due to a higher net charge of the polyelectrolyte. The corresponding molecular mechanism can be rationalized as follows. More counterions condense around the polyelectrolyte with higher salt concentrations in accordance with the dielectric decrement effects. Thus, the net charge of the polyelectrolyte is significantly reduced which lowers the ionic conductivity. At a specific salt concentration, the large number of condensed counterions results in strong repulsive interactions among the counterions which cannot be compensated by electrostatic attraction of the polyelectrolyte. In consequence for increasing salt concentrations, the amount of condensed counterions is reduced, which increases the effective net charge and thereby the ionic conductivity of the solution. In order to prove this conclusion, fixed values for the dielectric constant induce convergent values of $f_{\mathrm{cc}}\approx 0.73$ as well as $\Lambda /\Lambda_{0}=0.87$ for all concentrations $C^{1/2}\geq 0.15$ M${}^{1/2}$ (Figure 1). Hence, any non-linear change of the ionic conductivity for higher salt concentrations is absent, which is in direct contrast to previous experimental findings [65].

### 3.2. Molecular Properties of the Solvent: Donor/Acceptor Numbers and Chemical Hardnesses

**Figure 2:**
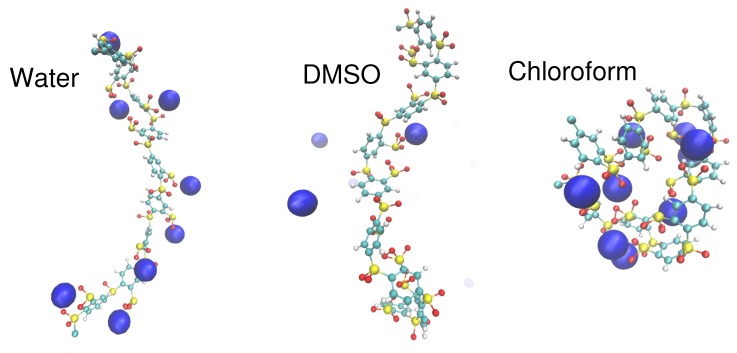
Simulation snapshots of sulfonated oligosulfonic acids with sodium counterions (blue spheres) in water (**left side**), dimethyl sulfoxide (DMSO) (**middle panel**), and chloroform (**right side**). Solvent molecules are ignored for the sake of clarity. Figure reproduced from Ref. [18].

The implications of the counterion condensation theory according to Equation (5) and Equation (13) highlight that solvents with large Bjerrum lengths reveal a higher tendency for condensed counterions and vice versa. In contrast to this assumption, recent experimental and atomistic MD simulation studies reported a more complex and non-unique behavior [15,18,70,71,72]. Thereby, it can be assumed that specific molecular interactions in terms of electronic and polarization contributions between the solvent molecules as well as the polyelectrolyte and the ions play a crucial role. For instance, atomistic MD simulations for short oligoelectrolytes as well as for rigid and artificial polyelectrolytes in various protic and aprotic solvents showed a condensation behavior which does not necessarily reveal a direct influence of the dielectric constant and thus the Bjerrum length [15,18]. Hence, it was shown that chemical concepts like donor numbers as well as polarization and dispersion interactions in combination with the explicit solvation interactions of the ions play a decisive role. Corresponding findings were also observed by coarse-grained MD simulations for simple bead-spring polyelectrolytes [70,71,72], where it was shown that the solvent affinity in combination with dispersion interactions crucially affect the condensation behavior as well as further macromolecular association mechanisms. In terms of visual inspection [18], the corresponding snapshots of sodium ions around highly charged sulfonated oligosulfonic acids in water, dimethyl sulfoxide (DMSO) and chloroform are shown in Figure 2. With regard to a simple consideration of the Bjerrum lengths, one would expect that the amount of condensed counterions increases from water ($\epsilon_{r}\approx 80$ and $\lambda_{B}\approx 0.7$ nm) via DMSO ($\epsilon_{r}\approx 47$ and $\lambda_{B}\approx 1.2$ nm) to chloroform ($\epsilon_{r}\approx 5$ and $\lambda_{B}\approx 11.2$ nm) [18].

In contrast, experimental outcomes [73] for a comparable system like in Ref. [18] and the results shown in Figure 2 reveal that a higher amount of sodium ions is condensed around polyelectrolytes in water when compared to DMSO and chloroform. With regard to these findings, it can be assumed that also specific interactions between the solvent molecules, the ions and the polyelectrolyte, respectively, modify the binding affinity of ions to the highly charged polyelectrolyte. These effects are usually ignored in standard mean field counterion condensation theories which highlights the crucial role of the solvent molcules for a reliable consideration.

**Figure 3:**
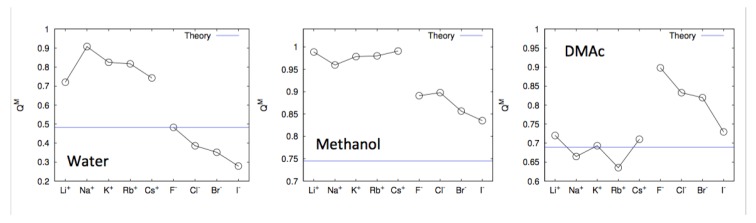
Fraction of condensed counterions around cylindric model polyelectrolytes with identical charge density in water (**left side**), methanol (**middle**) and dimethylacetamide (DMAc, **right side**). The straight blue lines correspond to the predicted fraction of counterions from counterion condensation theory. Figure reproduced from Ref. [15].

Further MD simulations revealed that the interaction energy between the coordinating solvent molecules and the ions has a massive influence on the dissociation behavior, and thus also on the amount of condensed counterions in combination with free solvation energies [15]. The authors performed atomistic MD simulations for rigid model polyelectrolyte systems in presence of counterions and different solvents water, methanol and N,N-dimethyl acetamide (DMAc). For a reasonable comparison, an improved version of the counterion condensation theory, namely the cell model PB approach [52] and the corresponding outcomes for the amount of condensed counterions were compared to the simulation findings. The corresponding values differ for various solvents and ions which rationalizes varying observations for the fraction of condensed counterions around model polyelectrolytes as shown in Figure 3.

As can be seen, the largest amount of condensed counterions around the cylindric polyelectrolytes can be observed for alkali ions like Li${}^{+}$, Na${}^{+}$, K${}^{+}$, Rb${}^{+}$ and Cs${}^{+}$ in methanol and water. In contrast, halide anions like F${}^{-}$, Cl${}^{-}$, Br${}^{-}$ and I${}^{-}$ reveal a significantly lower affinity for condensation. These findings are reversed for DMAc in which the anions reveal a stronger condensation behavior than the cations. Most remarkably, it becomes clear that some solvents promote cation over anion condensation and vice versa. With regard to these findings, one can conclude that molecular interactions besides pure electrostatic effects contribute significantly to the ion condensation behavior. In accordance, a more detailed study on the nature of the ion-solvent interactions revealed that dipolar electrostatic interactions are mainly responsible for the observed effects whereas entropic contributions in terms of solvent arrangements only account for 10–20% [15] to the ion free solvation energy, and are thus of minor importance [50]. Comparable findings were also reported for adiponitrile [74], in addition to propylene and ethylene carbonate [75], as well as for lithium salts in presence of urea [76,77].

In order to introduce a molecular rationale for the results shown in Figure 2 and Figure 3, it has to be noted that DMSO and water have two lone pair electrons, which imply a high nucleophilicity and thereby a favorable coordination of positively charged groups or cations. In consequence, most polar solvents reveal nucleophilic or electrophilic properties, which influence the ion solvation behavior and thus also the condensation affinities of ions around polyelectrolytes.

In order to categorize solvents with regard to their cation solvation properties, Gutmann et al. introduced the so-called empirical *donor number* (DN) scale [50,78,79], which accounts for the electron donating properties (donicity) of a solvent molecule. Vice versa, the electron-accepting properties (electrophilicty) of solvent molecules can be estimated from the Gutmann-Mayer acceptor number (AN) scale. Hence, a solvent which favors the solvation of cations has a high DN value whereas solvents which favor anion solvation usually reveal high AN values.

With regard to some experimental challenges associated with the measurement of DN and AN values, nucleo- or electrophilic properties of ion or molecular species can also be estimated by straightforward conceptual density functional theory (DFT) calculations [80]. Recent publications [80,81] suggested to interpret the affinity of solvents to specific solutes and ions as a consequence of the molecular properties. Thereby, solvation is considered as a specific chemical reaction which relies on the electronic polarization and charge transfer effects. In order to perform such an analysis, conceptual density functional theory calculations provide a simple, yet straightforward and reliable approach to estimate the strength of the electronic perturbation effects. Thus, the electronegativity of a molecule or ion is defined as [82]

(19) $$\chi =-{\left(\frac{\partial E}{\partial n}\right)}_{V}$$

with the total electronic energy functional *E* in combination with the number of electrons *n* under the constraint of a constant external or nuclear potential V. Moreover, the chemical hardness of a species, whose value can be interpreted as resistance against electronic changes, reads [82,83]

(20) $$\eta =\frac{1}{2}{\left(\frac{\partial^{2}E}{\partial n^{2}}\right)}_{V}=-{\left(\frac{\partial \chi }{\partial n}\right)}_{V}$$

which can be regarded as an inverse softness [83] and thus allows to discriminate between polarizable and less polarizable molecules [82]. In terms of a finite-difference approximation, the chemical hardness of a species can be estimated by [80,83,84]

(21) $$\eta ≃E_{\mathrm{HOMO}}-E_{\mathrm{LUMO}}$$

with the highest occupied and lowest unoccupied orbital energies $E_{\mathrm{HOMO}}$ and $E_{\mathrm{LUMO}}$. In consequence, species with small energy gaps between $E_{\mathrm{HOMO}}$ and $E_{\mathrm{LUMO}}$ are soft and thus easily polarizable and vice versa. In addition, the electronegativity of a species can be calculated in terms of the Mulliken definition

(22) $$\chi =-\frac{1}{2}\left(E_{\mathrm{HOMO}}+E_{\mathrm{LUMO}}\right)$$

which is a more straightforward expression when compared to Equation (19) [80].

Thus, for given ion- as well as polyelectrolyte-ion pairs with known electronegativities and chemical hardnesses, previous publications provided first expressions for suitable solvent-ion pairs in order to foster ion dissociation [80,81]. As was discussed in more detail in Ref. [80], it can be shown that ion dissociation is least favorable for solvents that have electronegativity values of

(23) $$\chi_{S}^{\max}=\frac{\chi_{A}\left(\eta_{B}+\eta_{S}\right)+\chi_{B}\left(\eta_{A}+\eta_{S}\right)}{\eta_{A}+\eta_{B}+2\eta_{S}}.$$

which results in

(24) $$\chi_{S}^{\max}=\frac{1}{2}\left(\chi_{A}+\chi_{B}\right)$$

for identical chemical hardnesses $\eta_{A}=\eta_{B}=\eta_{S}$. The corresponding results reveal that ion dissociation is favored for strongly acidic or basic solvents. However, if the solvent has a comparable affinity to anions and cations such that the solvation energies of both ions are roughly comparable, a high condensation behavior can be observed instead. In close agreement, it was discussed in Ref. [81], that most stable ion complexes can be observed for comparable electronegativity differences between the cationic species and the solvent as well as the anionic species and the solvent, respectively. A more detailed discussion of basic mechanisms can be found in Refs. [80,81].

**Figure 4:**
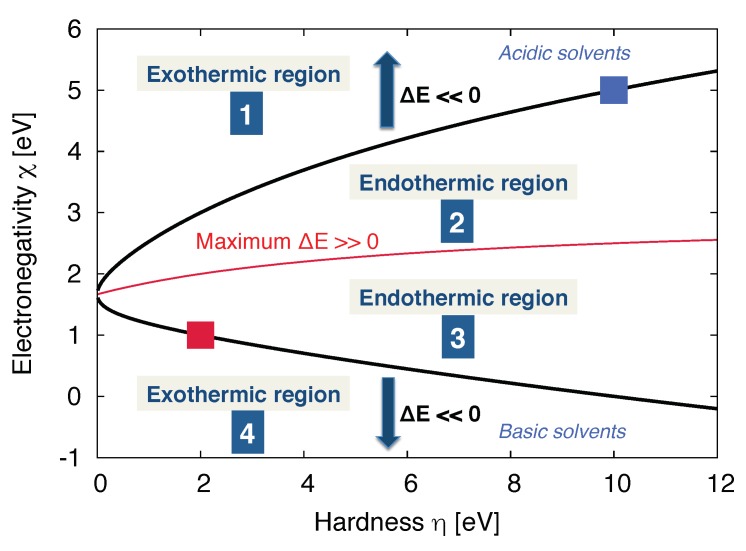
Endothermic $\Delta E_{\mathrm{AB}}>0$ and exothermic $\Delta E_{\mathrm{AB}}<0$ regions for solvents with distinct hardnesses $\eta_{S}$ and electronegativities $\chi_{S}$ in combination with a cation (blue square) with arbitrary values of $\eta_{A}=10$ eV and $\chi_{A}=5$ eV and an anion with arbitrarily chosen values of $\eta_{B}=2$ eV and $\chi_{B}=1$ eV (red square). The red solid line denotes the maximum value for an endothermic reaction energy as defined for a solvent with $\chi_{S}^{\max}$ (Equation (23)). The black solid lines denote the separatrices for values of $\Delta E_{\mathrm{AB}}=0$. Figure reproduced from Ref. [80].

For an arbitrarily chosen polyelectrolyte-ion pair with given electronegativities as well as hardnesses, the corresponding regions for acidic and basic solvents as well as the regions for endothermic (counterion condensation) and exothermic reactions (counterion dissociation) are shown in Figure 4.

In summary, the corresponding results reveal that the molecular nature of the solvent crucially affects the counterion condensation behavior in terms of polarization and charge transfer effects. In contrast to previous theoretical considerations for continuum solvents, recent experimental and simulation results imply a more complex behavior which relies on the molecular properties of the involved species. Thus, counterion dissociation is strongly favored if the individual chemical properties like electronegativities and hardnesses of the solvent and the charged species differ significantly. Besides all molecular interactions, it has to be noted that electrostatic interactions remain the main driving force for all ion condensation and dissociation effects, such that the aforementioned chemical properties are only responsible for the observed slight differences among the ions (Figure 3).

### 3.3. Weak Polyelectrolytes: pH Value Effects

**Figure 5:**
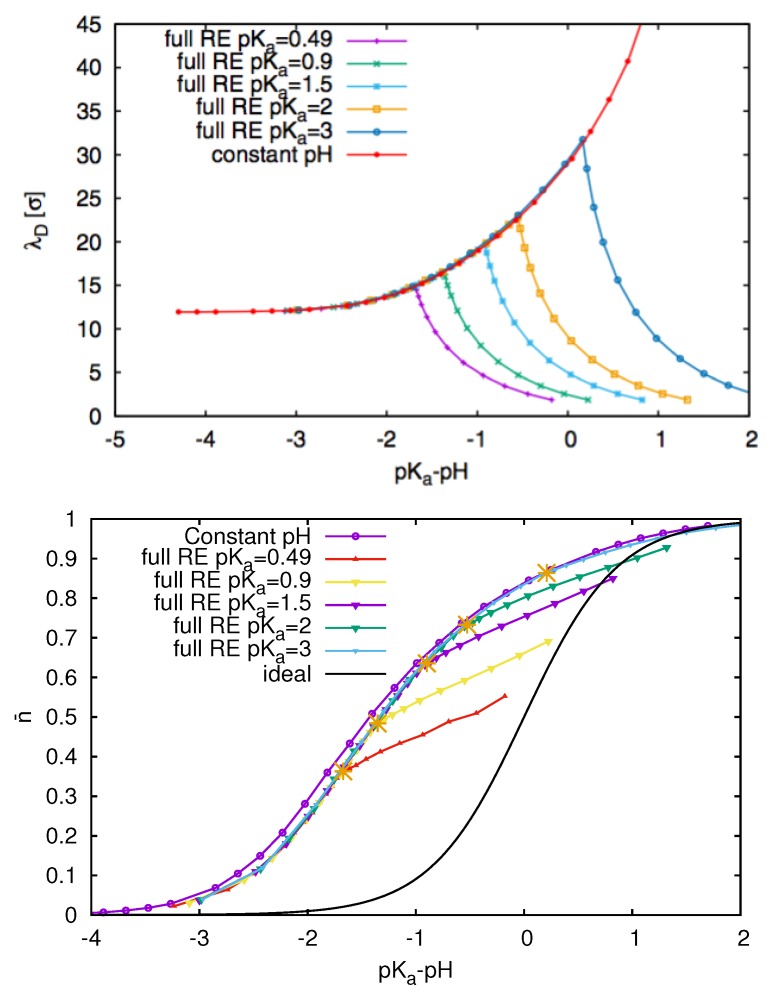
Resulting Debye-Hückel lengths $\lambda_{D}$ and degree of association $\bar{n}$ (**bottom**) for flexible weak polyelectrolytes with different pK${}_{a}$ values and a fixed Bjerrum length as obtained by the reaction ensemble (RE) method and the constant pH method. The actual pH value of the solution is defined by the relation pK${}_{a}$-pH. Figures reproduced from Ref. [24].

In addition to strong polyelectrolytes like DNA or poly(styrene sulfonate) (PSS) which reveal a high degree of ionization in water, weak polyelectrolytes like polyacrylic acid (PAA) are often not fully dissociated in aqueous solution, such that some titrable molecular groups remain uncharged. These effects can mainly be observed for polyacids where the dissociation behavior of hydronium ions (protons) is strongly influenced by the surrounding pH value. Hence, the degree of ionization crucially depends on the pH value of the solution and is thus a function of the considered solvent. From a theoretical point of view, it is a challenging task to include pH dependent dissociation mechanisms in simulations [24,25]. An often used modelling approach is the so-called constant pH method [85,86,87], which assumes a low number density of the dissociated protons such that the pH value of the solution does not change significantly upon dissociation. Despite the computational efficiency of the constant pH method, a more reliable approach with regard to electrostatic screening effects is the reaction ensemble approach [24,25,88,89]. The impact of the resulting charge screening effects can be seen in Figure 5 which depict the results of the constant pH and the reaction ensemble method. In more detail, each polyelectrolyte is defined by a pK${}_{a}$ value which corresponds to the logarithmic chemical equilibrium constant in accordance with the proton dissociation-association reaction. Thus, if the pH value of the solution is lower than the pK${}_{a}$ value of the polyelectrolyte with pK${}_{a}$-pH > 0, the polyelectrolyte and the protons remain associated and the net charge is low. The missing presence of explicit protons in the solution in the constant pH method induces spurious deviations in the resulting Debye-Hückel screening length $\lambda_{D}=1/\kappa_{D}$ in agreement with Equation (4). Noteworthy, the amount of charged groups $\bar{n}$ is significantly smaller in the reaction ensemble method which also can be attributed to electrostatic screening effects.

Hence, the corresponding results reveal that electrostatic screening effects are also important for weak polyelectrolytes which show a pH dependent dissociation behavior. In consequence, the dissociation of protons as well as ions strongly influences the properties of the polyelectrolyte and the amount of charged groups.

## 4. Specific Ion Effects

For a long time, it is known that ions differ crucially in their tendency for ion pair formation. With regard to this point, some ion pairs are more stable than others which also depends on the chosen solvent and further components of the solution. Corresponding experimental and simulational approaches suggested that this behavior is universal and occurs for all ions in aprotic as well as protic solvents due to different solvation interactions [18,20,34,74,90,91,92]. Previous explanations for these effects often cite the *law of matching water affinities* (LMWA), which states that ion pairs with comparable water affinities reveal the highest ion pair formation stabilities [93]. In more detail, ions which are kosmotropes, meaning that they are water-structure makers, and ions which are chaotropes (water-structure breakers) form most stable ion pairs with their cationic or anionic counterparts. This conclusion is further supported by positive solvation enthalpies for kosmotropic-kosmotropic as well as chaotropic-chaotropic ion pairs in aqueous solution [93,94]. As already mentioned, recent publications revealed that these effects are also evident for aprotic solvents and it can be expected that comparable effects influence the condensation or dissociation affinities of counterions around polyelectrolytes [15,34,92,95].

With regard to this point, atomistic and coarse-grained MD simulations were often used to study the distribution of various counterion species around polyelectrolytes in aqueous solution [15,54,55,70,71,72]. Thereby, the authors studied the spatial concentration of various alkali and halide ions around rigid and stretched polyelectrolytes like polyglutamic acid, polystyrene sulfonate, polyallylamine hydrochloride and polyacrylic acid which also allows to study the influence of the polyelectrolyte geometry on the condensation behavior [54,55]. Further studies also focused on coarse-grained MD simulations in order to unravel the role of flexible polyelectrolytes on the association properties [71,72]. Thus, all studies point out that counterion condensation mechanisms rely on the molecular and electronic properties of ions as well as the molecular geometry of the polyelectrolyte. For instance, the local counterion density $\rho_{+}\left(r\right)$ and the integrated fraction $x_{+}$ of condensed counterions

(25) $$x_{+}\left(r\right)=\frac{2\pi }{N_{+}}L\int_{r_{0}}^{r}\rho_{+}\left(r^{\prime }\right)r^{\prime }dr^{\prime }$$

with the length of the simulation box *L* and the number of counterions N${}_{+}$ may show significant differences between different cations (Na${}^{+}$, K${}^{+}$, and Cs${}^{+}$) and an artificial polyelectrolyte chain [54]. As it was observed, more sodium ions are condensed at the polyelectrolyte when compared to K${}^{+}$ or Cs${}^{+}$. Thus, the dissociation tendencies of the cations increase with their ionic radii which can be brought into agreement with varying chemical hardnesses as well as electronegativity values in accordance with Equations (19) and (20).

Although a reasonable agreement between the modified PB theory (Equation (15) and the simulation results can be observed at large distances, significant deviations become evident at short scales. However, it has to be noted that the hydration potential as well as the modified PB equation miss any reliable physical interpretation which underpins the fact that our theoretical understanding of short-range attraction mechanisms is still very limited. Moreover, the corresponding values show non-linear variations among the deviations for the cations and thus should only be interpreted as more or less beneficial fitting parameters without a fundamental meaning. Despite its heuristic properties, the hydration potential as well as the modified PB equation imply that the solvent plays a decisive role in the counterion condensation behavior. Thus, all assumptions point to complex interactions between the ions, the solvent and the polyelectrolyte which complicates the development of straightforward mean field electrostatic descriptions.

**Figure 6:**
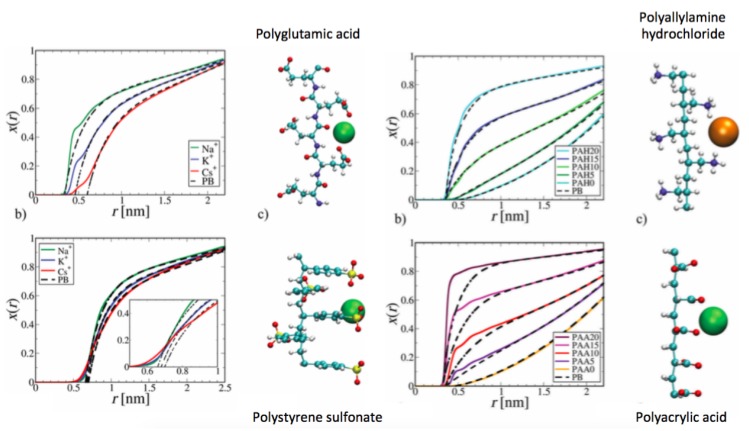
Fraction of condensed counterions x(r) around polyglutamic acid (**top left**), polyallylamine hydrochloride (**top right**), polystyrene sulfonate (**bottom left**) and polyacrylic acid (**bottom right**) for various counterion species as denoted in the legend. The dashed black lines correspond to the fits of the modified PB equation (Equation (15). The effects of varying line charge density are studied for polyacrylic acid and polyallylamine hydrochloride on the right side. Figure adapted from Ref. [55].

In addition to the properties of the counterions, it also has to be noted that the corresponding molecular nature of the polyelectrolyte induces some distinct effects on the counterion distribution [55]. The corresponding fractions of condensed counterions around various polyelectrolytes are shown in Figure 6.

As can be seen, the fraction of condensed counterions differs significantly for the considered polyelectrolytes polyglutamic acid, polyallylamine hydrochloride, polystyrene sulfonate (PSS) and polyacrylic acid. With regard to visual inspection, it becomes clear that the differences mainly arise due to variations of the polyelectrolyte geometry, line charge density as well as the molecular properties of the charged polyelectrolyte groups. As a specific example, flexible side chains with highly charged groups like in PSS establish a strong coordination of the counterions, while weaker condensation effects can be observed for more rigid charge groups like in polyglutamic acids. With regard to the discussions in the previous subsection, it can be also assumed that the charged groups differ in their counterion affinity due to varying electrophilic and nucleophilic behavior. It thus becomes clear that the respective simulation findings highlight significant deviations to previous theoretical predictions which rely on homogeneous and linear charge distribution as well as the neglect of molecular details.

As already mentioned, also changes in the line charge density induce higher order variations for the amount of condensed counterions (right side of Figure 6). With regard to the modified PB equation (Equation (15)), it becomes evident that even the use of the hydration potential does not correct for significant deviations observed for high line charge densities at short distances [55]. Interestingly, the differences to the modified PB theory are less pronounced for polyallylamine hydrochloride whereas the line charge density specfically at short distances reveals significant changes for polyacrylic acid. Furthermore, the authors of Ref. [55] studied distinct degrees of ionization for polyacrylic acid and polyallylamine hydrochloride as denoted by the numbers in the legend. Hence, a PE-20 value corresponds to a degree of 100% ionization, a PE-15 value corresponds to 75% ionization, PE-10 value corresponds to 50% ionization, a PE-5 value corresponds to 25% ionization, and a PE-0 value corresponds to 0% ionization. As can be seen, the deviations of polyacrylic acid to the PB equation are more pronounced for high degrees of ionization, thereby showing that the hydration potential is best fitted for negligible degrees of ionization. Corresponding conclusions can be drawn for polyallylamine hydrochloride where the modified PB equation provides a reasonable level of accuracy in order to match the simulation outcomes. Hence, it can be concluded that the empirical properties of the modified PB equation are mainly valid for low and moderate line charge densities which highlights the mean field behavior of the approach. In summary, the simulation findings imply that the counterion distribution and the number of condensed counterions depend crucially on the considered ion and polyelectrolyte species. A deeper understanding of the molecular properties as well as further interaction mechanisms is of sufficient need in order to improve our current knowledge.

In further publications, it also was shown in Ref. [96] that differences in the monomeric charge state crucially affect the counterion condensation behavior as well as the attraction of oppositely charged macromolecules. The corresponding effects can be attributed to polyelectrolyte complex coacervation mechanisms which correspond to a liquid-liquid phase separation mechanism driven by electrostatic attraction [97]. Closely related, also proteins and polyelectrolytes show a like-charge complexation mechanism which is mainly influenced by the distribution and the amount of charged patches on the protein. Further analysis revealed a pronounced correlation between the potentials of mean force in combination with protein orientation effects as well as the number of dissociated counterions [98]. In addition, certain weakly charged polymers also show transitions from swollen to collapsed conformations and vice versa. At low salt concentrations, ion-screening and bridging effects prefer collapsed conformations whereas ion-specific direct interactions induce swollen conformations at high salt concentrations [99]. Noteworthy, specifically multivalent salt solutions are known to favor collapsed polyelectrolyte conformations. In more detail, multivalent counterions are strongly attracted and thus screen the monomeric charge due to their valency more effectively [100]. In contrast, repulsion effects between polyelectrolytes were attributed to overcharging as well as ionic confinement mechanisms [101]. In contrast to bulk solutions, polyelectrolytes in confinement and in presence of dielectric mismatch conditions show a transitional behavior into various conformations. Here, surface polarization mechanisms as induced by a dielectric mismatch in combination with the electrostatic interactions between the monomers and multivalent counterions favor changes in the conformational behavior when compared to bulk conditions [102]. The corresponding findings reveal that specifically also the conformational properties of the polyelectrolyte are modified by distinct charge states, multivalent ions, dielectric mismatch conditions as well as the presence of confinement effects and high salt and polyelectrolyte concentrations.

## 5. Co-Solute and Co-Solvent Effects

In addition to solvent as well as specific ion effects, it has to be noted that further components of the solution, i.e., co-solutes or co-solvents, also have a significant influence on the degree of counterion condensation as well as the amount of stable ion pairs. The outcomes of atomistic MD simulations for various ion and solvent species as well as solvent mixtures suggested that specifically the binding behavior of the solvent influences the degree of ion association [19,20,34,103]. Depending on the composition of the mixture, one can define a main solvent and a co-solvent or co-solute. In terms of a molecular rationale, it was shown that the binding behavior of the co-solvent to the ions or the polyelectrolyte influences the fraction between bound and free ions. Whenever the co-solvent replaces solvent molecules around the ions or the polyelectrolyte in terms of a preferential binding behavior, the chemical equilibrium between free and bound ions is shifted to the dissociated state. In contrast, if the co-solvent molecules are preferentially excluded, the chemical equilibrium is shifted to the bound state [19,20,34,103]. The corresponding observation can be brought into agreement with the Kirkwood-Buff theory of solutions, with the preferential binding coefficient as defined by

(26) $$\nu =N_{\mathrm{cs}}-\frac{\rho_{s}}{\rho_{\mathrm{cs}}}N_{s}$$

with the density $\rho_{\mathrm{cs}}$ and number $N_{\mathrm{cs}}$ of co-solvent and the density $\rho_{s}$ and number $N_{s}$ of solvent molecules, respectively [103,104,105]. Thus, a value of ν>0 corresponds to a preferential binding behavior whereas ν<0 indicates a preferential exclusion behavior of the the co-solvent around the considered species. With regard to this definition, the differences in the amount of co-solvent molecules around the free (subscript F) and bound (subscript B) ions as central species in accordance with $\Delta \nu =\nu_{F}-\nu_{B}$ thus determine the chemical equilibrium of the corresponding ion association-dissociation reaction. Specifically for low co-solvent concentrations, the chemical equilibrium constant in presence of co-solvent species can be simplified according to

(27) $$K\approx K_{0}\exp\left(\Delta \nu \right)$$

which implies that a preferential binding behavior with positive preferential binding coefficients fosters the dissociation of the counterions and vice versa [20,34].

Corresponding atomistic MD simulations validated the predicted behavior for sodium chloride ion pairs in water/DMAc [20] and water/DMSO mixtures [19]. In terms of a detailed molecular explanation, the non-ideal distribution of co-solvent and solvent species around charged objects like ions or polyelectrolytes crucially affects the corresponding chemical potentials and thus the chemical equilibrium constant for the considered solvation reaction. With regard to previous discussions, these inhomogeneities can be related to strongly varying interaction energies and hence chemical activities of the involved species such that higher order effects become more dominant. In consequence, the ion dissociation of polyelectrolytes changes significantly in presence of multi-component solutions when compared to homogeneous media.

**Figure 7:**
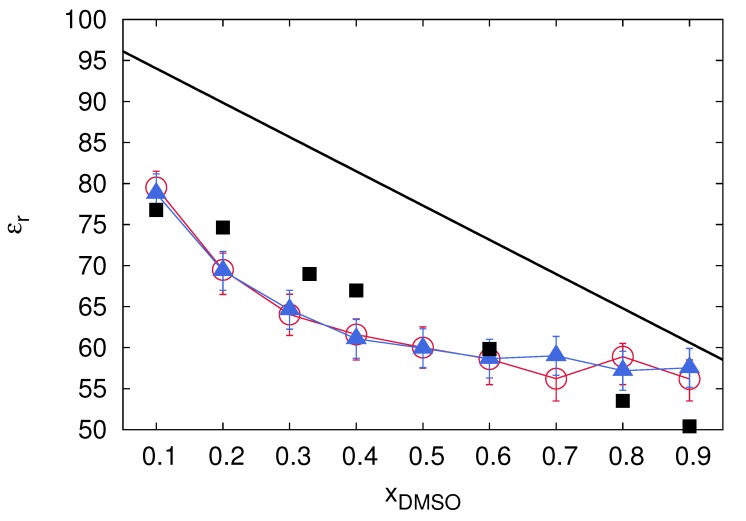
MD simulation outcomes of the resulting dielectric constant $\epsilon_{r}$ for an aqueous DMSO solution with increasing mole fractions of DMSO $x_{\mathrm{DMSO}}$ in presence (blue triangles) and absence of low concentrated ion pairs (bue). The corresponding values for TIP3P water and DMSO are $\epsilon_{r}^{\mathrm{TIP}3P}=95.32$ and $\epsilon_{r}^{\mathrm{DMSO}}=55.54$. The black squares correspond to experimental results. Figure reproduced from Ref. [19].

Furthermore and in close agreement with the previously discussed dielectric decrement effects, the presence of uncharged co-solvent molecules also induces global variations of the dielectric constant. For a mole fraction of the co-solvent species $x_{\mathrm{cs}}$ in a binary solution, the ideal dielectric constant of a homogeneous solution reads

(28) $$\epsilon_{r}^{\mathrm{sol}}=\epsilon_{r}^{s}\left(1-x_{s}\right)+\epsilon_{r}^{cs}x_{s}$$

which shows a linear variation between the individual dielectric constants $\epsilon_{r}^{s}$ and $\epsilon_{r}^{cs}$ of the pure solvent or co-solvent solution, respectively. Hence, with increasing mole fraction of the co-solvent, one usually observes a change of the dielectric constant from the value for the pure solvent towards the dielectric constant of the pure co-solvent. In terms of highly polar aqueous solutions, most often a decrease of the dielectric constant can be observed for increasing co-solvent concentrations which strengthens electrostatic interactions between charged species and hence a stronger counterion condensation behavior for polyelectrolytes. Comparable conclusions can be also drawn with regard to semi-coarse-grained MD simulations with the MARTINI force field and for concentrated polyelectrolyte solutions, high salt conditions as well as dense polyelectrolyte solutions [33,59]. However, with regard to the fact that most binary solutions reveal non-ideal effects due to the presence of clustering tendencies, a non-linear variation of the dielectric constant can be observed instead (Figure 7).

The corresponding outcomes of MD simulations for water and DMSO highlight the variation of the dielectric constant in presence as well as in absence of low concentrated ion pairs. Hence, one can observe a dielectric decrement effect with a strongly non-linear variation of the dielectric constant in terms of increasing DMSO concentrations.

## 6. Summary and Conclusions

In this short review article, we highlighted the crucial role of the solvent and the ion species on the dissociation as well as on the conformational behavior of the polyelectrolyte. Previous literature results revealed that simple and straightforward electrostatic mean field descriptions are not sufficient for a detailed prediction of the observed behavior. Even extensions of the PB equation which include empirical corrections often reveal deviations to experimental and simulation findings for moderately and highly charged polyelectrolytes.

Hence, one can conclude that the molecular properties of the solvent, the polyelectrolyte and the ion species are of significant importance in order to understand the observed effects. With regard to this point, recent theoretical approaches concentrated on molecular properties like electronegativities, chemical hardnesses as well as charge transfer mechanisms in order to provide a first step towards a more fundamental understanding of the observed effects and solvation mechanisms. The corresponding affinity between the solvent molecules and the charged species influences the binding energy and hence the dissociation or condensation behavior. Moreover, also the presence of co-solvent species in mixtures crucially affect the dissociation behavior in terms of non-ideal mixing effects besides variations of the dielectric constant.

In summary, it can be concluded that our current molecular understanding of these effects is rather low. Most of the underlying mechanisms can be related to higher order effects such that straightforward and simple mean field approaches may not be fully applicable for a reasonable description of the corresponding observations. In consequence, more effort has to be spent in order to unravel the behavior of polyelectrolytes and their ions in various protic and aprotic solvents, respectively, or their mixtures. As a beneficial side effect, such refined theories may also help to improve technological applications in the future.
